# Diameter of ureteral access sheath and ureteral stenosis development: a systematic review

**DOI:** 10.1590/acb387423

**Published:** 2023-10-30

**Authors:** Tamires Battistini Pissaia, Mikhael Belkovsky, Carlo Camargo Passerotti, Everton Luiz de Almeida Artifon, Jose Pinhata Otoch, José Arnaldo Shiomi da Cruz

**Affiliations:** 1 Universidade Nove de Julho – Urology Department – São Paulo (SP) – Brazil.; 2 Universidade de São Paulo – Medical School – Surgical Technique Department – São Paulo (SP) – Brazil; 3 Hospital Alemão Oswaldo Cruz – Prostate Institute – Urology Department – São Paulo (SP) – Brazil.

**Keywords:** Constriction, Ureteral Calculi, Ureteroscopy

## Abstract

**Purpose:**

Ureteral access sheaths (UAS) are widely used in ureteroscopy. UAS are believed to pose a significant risk for ureteral stenosis due to ureteral mucosal compression, but little evidence supports this claim. Our systematic review aimed to investigate the relationship between different UAS diameters and stenosis risk.

**Methods:**

A systematic search was conducted in PubMed, Embase, Web of Science, Scopus, and Cochrane, from its inception to May 2023. Preferred Reporting Items for Systematic Reviews and Meta-Analyses (PRISMA) and Cochrane guidelines were followed. χ^2^ test was performed to compare the prevalence within the groups.

**Results:**

Six nonrandomized trials and one randomized, with a total of 962 patients, were included. The overall incidence of ureteral stenosis of 0.9%. UAS sizes were: 9.5/11.5Fr, 10/12Fr, 11/13Fr, 12/14Fr, and 14/16Fr. Within each subgroup, the incidence of ureteral stenosis was: 0.4, 8, 0, 1, and 1% (p = 0.099). No trend for stenosis was observed among larger UAS.

**Conclusions:**

In this systematic review, no relationship between UAS diameter and incidence of ureteral stenosis was observed. Nonetheless, additional randomized controlled trials are required to support this finding.

## Introduction

The ureteral access sheath (UAS) is widely employed in ureteroscopy, facilitating repeated insertion of the ureteroscope into the upper ureter without the need for a working wire^1^. This tool has shown to reduce operative time and costs^2^.

As studies observed a reduced ureteral blood blow in animal models^3^, it is believed that UAS usage may heighten the risk of ureteral strictures due to reduced ureteral perfusion during the procedure. However, clinical studies have yielded conflicting results regarding this risk^3^^–^ 5.

In humans, it has been demonstrated that ureteral stenosis may arise from procedural complications, prolonged use of the ureteral sheath, kidney stones, inflammation, urinary tract infections, and trauma. Age and sex are not related with the risk of ureteral strictures^6^.

The objective of this study was to investigate whether the use of larger UAS is associated with ureteral stenosis in patients undergoing the procedure.

## Methods

### Eligibility and data extraction

We restricted our analysis to studies that met all the following inclusion criteria:

- Patients undergoing endourological procedures with ureteral sheaths of various diameters;
- Studies that evaluated the incidence of ureteral stricture;
- Follow-up time of at least one month.

Our exclusion criteria included:

- Review articles;
- Editorial responses.

### Search strategy and registration

We conducted a systematic review of the literature in the following databases: PubMed, Embase, Web of Science, Scopus, and Cochrane in May 2023. Our study was registered with PROSPERO (CRD42023418440).

The search strategy was as follows: (stricture OR strictures OR injury OR injuries) AND (“ureteral sheath” OR “ureteral access sheath” OR “ureteral access sheaths”). Additionally, we manually reviewed the reference lists of all studies, meta-analyses, and reviews.

*Screening and data collection*

Two authors (TBP and MB) independently screened and extracted data based on predefined search criteria and quality assessment methods. Any disagreements between authors were solved with the involvement of the third author (JASC).

### Risk of bias, quality assessment, and statistics

Each study was analyzed in accordance with Cochrane recommendations to assess the risk of bias for both randomized and non-randomized studies. We conducted a simple analysis of the categorical variable of presence vs. absence of ureteral stenosis in each subgroup and compared them using the χ^2^ statistic.

## Results

**Figure 1:**
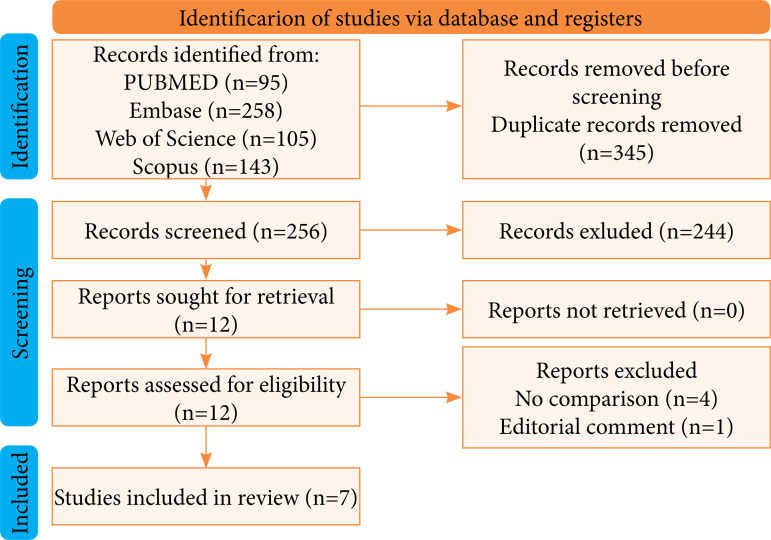
Percentage of ureteral stricture by sheath diameter.

Source: Elaborated by the authors.

Six nonrandomized trials^3^^,^ 4 ^,^ 7 ^,^ 8 and one randomized controlled trial^5^ were included, involving a total of 962 patients. The Preferred Reporting Items for Systematic Reviews and Meta-Analyses (PRISMA) workflow is presented in Fig. 1. The male population ranged from 41.3 to 62.4%. The mean age was 53 years old, with average follow-up time of 15 months, ranging from six weeks to 68 months (Table 1).

**Table 1:** Baseline characteristics of included studies.

Authors	Design	Groups (French)	Patients	Male (%)	Mean Age (years)	Operative time (min): G1/G2	Calculation size: G1/G2	Complications (%): G1/G2	Follow-up time (months)
Rodrigues and Einser^1^^§^	NRCT	5 groups	157	56.0	61	NA	NA	NA	12
Delvecchio et al.^3^^§^	NRCT	G1:10/12 G2:12/14 G3:14/16	71	60.5	45	NA	NA	NA	3
Shvero et al.^4^	NRCT	G1:9,5/11,5 G2:12/14	165	62.4	56	39/61	23/34	11/12	4
Aykanat et al.^5^	RCT	G1:9,5/11,5 G2:12/14	320	55.9	48	54/53	16/15	5/12	12
Tracy et al.^7^	NRCT	G1:12/14 G2:14/16	168	41.3	54	72/63	102/146	11/11	1,5
Jordan et al.^8^^§^	NRCT	G1:12/14 G2:14/16	237	55.3	54	NA	NA	NA	12
Breen 2021^10^^§^	NRCT	G1:10-14 G2:14/16	355	50.7	55^†^	NA	NA	6/4	68

§ Conference abstracts;

† median;

RCT: randomized controlled trial; NRCT: no randomized controlled trial. Source: Elaborated by the authors.

**Figure 2:**
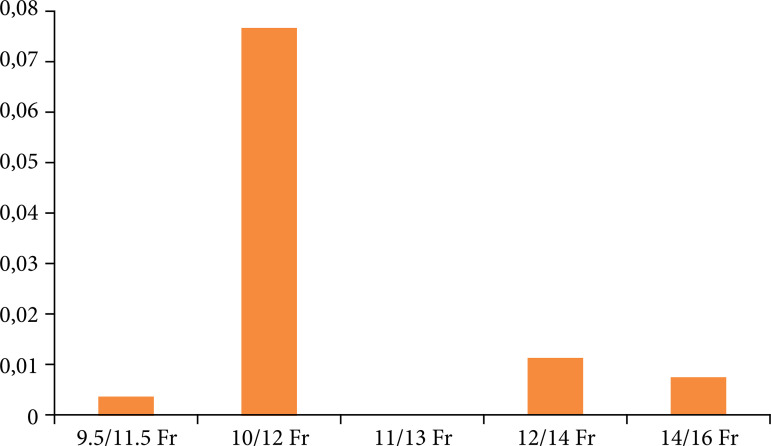
Percentage of ureteral stricture by diameter. No increased incidence of ureteral stenosis was observed with larger ureteral access sheath.

Source: Elaborated by the authors.

Assessing the available pairwise comparisons within the studies, larger UAS present with equal or greater operative time, similar or greater calculation size. Figure 2 displays the number of patients in each subgroup and the percentage of stenosis based on the ureteral sheath diameter. A χ^2^ test was conducted to compare the prevalence of ureteral stenosis across the subgroups, indicating independence among the groups with p = 0.099.

Table 2 shows the distribution of the ROBBINS-I assessment of the nonrandomized included studies. Confounding variables related to different intraoperative assessments of ureteral diameter may interfere with UAS size selection, and the patient selection process was poorly described. Meta-analysis was not conducted due to the non-comparability of study groups with different UAS sizes among cohorts.

**Table 2:** Risk of bias in nonrandomised studies of interventions of included studies.

	Confounding	Selection	Classification	Deviations	Missing data	Measurement	Reported results	Overall
Rodrigues and Einser^1^	Serious	Moderate	Low	Low	Serious	Low	Moderate	Serious
Delvecchio et al.^3^	Serious	Serious	Low	Low	No information	Low	Moderate	Serious
Shvero et al.^4^	Moderate	Moderate	Low	Low	Moderate	Low	Low	Moderate
Tracy et al.^7^	Moderate	Moderate	Low	Low	Moderate	Low	Low	Moderate
Jordan et al.^8^	Critical	Serious	Low	Low	Serious	Low	Moderate	Critical
Breen 2021^10^	Serious	Serious	Low	Low	No information	Low	Moderate	Serious

Source: Elaborated by the authors.

## Discussion

In this systematic review comparing different UAS diameters, no increase of ureteral strictures with larger UAS sizes was observed. The overall incidence of ureteral strictures was 0.9%.

The only randomized controlled trial reported a similar incidence of ureteral stricture in the 12/14 Fr UAS group compared to the 9.5/11.5 Fr UAS group (2.5% versus 0.6%, p = 0.37)5. Among nonrandomized trials, even considering that larger UAS sizes have equal or greater operative time, calculation size, there was no increase of ureteral stenosis with larger UAS. Indeed, it was previously observed that the degree of ureteral lesions does not correlate with the incidence of stenosis^9^.

A previous noncomparative study using 12/14 Fr UAS observed a ureteral stricture incidence of 1.8% during a 38-week follow-up, which aligns with our overall incidence^9^. One interesting finding was the higher incidence in the 10/12 Fr group. We hypothesize that this finding may be a statistical artifact caused by the small number of patients (only 13), with one occurrence of ureteral stenosis^3^.

It has been suggested that UAS may contribute to the development of ureteral strictures, which has raised concerns about using larger UAS sizes, despite their technical advantages in reaching challenging calculi^1^. Our study challenges this theory and advocates for a more liberal use of larger UAS.

This study has limitations:

- The heterogeneity of baseline characteristics between the groups and absence of more granular data, that challenges pooled quantitative analyses;
- Short follow-up of some of the included studies;
- Lack of sheath time.

The development of ureteral strictures following ureteroscopy appears to be a rare and unexplained phenomenon. Our findings suggest that it is not associated with UAS size. However, open questions remain, including whether our findings would be confirmed in additional randomized controlled trials and which variables could predict this complication.

## Conclusion

In this systematic review, no relationship between UAS diameter and incidence of ureteral stenosis was observed. Nonetheless, additional randomized controlled trials are required to support this finding.

## Data Availability

The data will be available upon request
